# Quantification of Carbonic Anhydrase Inhibitors and Metabolites in Urine and Hair of Patients and Their Relatives

**DOI:** 10.3390/biology11101379

**Published:** 2022-09-21

**Authors:** Alfredo Fabrizio Lo Faro, Anastasio Tini, Giulia Bambagiotti, Filippo Pirani, Andrea Faragalli, Flavia Carle, Elena Pacella, Artan Ceka, Marco Moretti, Massimo Gottardi, Nicola Vito Lassandro, Michele Nicolai, Marco Lupidi, Cesare Mariotti, Francesco Paolo Busardò, Jeremy Carlier

**Affiliations:** 1 Unit of Forensic Toxicology, Section of Legal Medicine, Department of Biomedical Sciences and Public Health, Marche Polytechnic University, Via Tronto 10/a, 60126 Ancona, AN, Italy; 2 Center of Epidemiology Biostatistics and Information Technology, Department of Biomedical Sciences and Public Health, Marche Polytechnic University, Via Tronto 10/a, 60126 Ancona, AN, Italy; 3 Department of Sense Organs, Faculty of Medicine and Dentistry, Sapienza University of Rome, Viale del Policlinico 155, 00161 Rome, RM, Italy; 4 Department of Laboratory Medicine, Reunited Hospitals Torrette of Ancona, Via Conca 71, 60126 Ancona, AN, Italy; 5 Comedical srl, Via della Cooperazione 29, 38123 Trento, TN, Italy; 6 Eye Clinic, Marche Polytechnic University, Via Tronto 10/a, 60126 Ancona, AN, Italy

**Keywords:** carbonic anhydrase inhibitor, dorzolamide, brinzolamide, acetazolamide, case report, urine, hair, liquid chromatography-tandem mass spectrometry

## Abstract

**Simple Summary:**

Carbonic anhydrase inhibitors such as dorzolamide, brinzolamide, and acetazolamide are prescription drugs prohibited in sports. Detecting these substances and their biomarkers of consumption in urine and hair is crucial to documenting misuse in doping. We quantified dorzolamide, brinzolamide, acetazolamide, and their metabolites in the urine and hair of 88 patients under treatment, and samples of the patients’ relatives were analyzed to assess potential for accidental exposure. We found that cutoff concentrations of urinary dorzolamide and brinzolamide are necessary to preclude false positives due to contamination or passive exposure. Additionally, we reported the first concentrations of brinzolamide in hair.

**Abstract:**

Carbonic anhydrase inhibitors (CAIs) are prescription drugs also used in doping to dilute urine samples and tamper with urinalyses. Dorzolamide, brinzolamide, and acetazolamide are prohibited by the World Anti-Doping Agency. Detecting CAIs and their metabolites in biological samples is crucial to documenting misuse in doping. We quantified dorzolamide, brinzolamide, acetazolamide, and their metabolites in the urine and hair of 88 patients under treatment for ocular hypertension or glaucoma. Samples of the patients’ relatives were analyzed to assess potential for accidental exposure. After washing, 25 mg hair was incubated with an acidic buffer at 100 °C for 1 h. After cooling and centrifugation, the supernatant was analyzed by ultra-high-performance liquid chromatography-tandem mass spectrometry (UHPLC-MS/MS). Urine (100 μL) was diluted and centrifuged before UHPLC-MS/MS analysis. Run time was 8 min through a reverse-phase column with a mobile phase gradient. MS/MS analysis was performed in a multiple-reaction monitoring mode after positive electrospray ionization. Median urinary concentration was 245 ng/mL (IQR: 116.2–501 ng/mL) for dorzolamide, 81.1 ng/mL (IQR: 35.9–125.3 ng/mL) for *N*-deethyl-dorzolamide, 0.77 ng/mL (IQR: 0.64 ng/mL–0.84 ng/mL) for *N*-acetyl-dorzolamide, 38.9 ng/mL (IQR: 20.4–79.2 ng/mL) for brinzolamide, and 72.8 ng/mL (IQR: 20.7–437.3 ng/mL) for acetazolamide. Median hair concentration was 0.48 ng/mg (IQR: 0.1–0.98 ng/mg) for dorzolamide, 0.07 ng/mg (IQR: 0.06–0.08 ng/mg) for *N*-deethyl-dorzolamide, 0.40 ng/mL (IQR: 0.13–1.95 ng/mL) for brinzolamide. Acetazolamide was detected in only one hair sample. Dorzolamide and brinzolamide were detected in the urine of three and one relatives, respectively. Cutoff concentrations of urinary dorzolamide and brinzolamide are necessary to preclude false positives due to contamination or passive exposure. We reported the first concentrations of brinzolamide in hair.

## 1. Introduction

The carbonic anhydrase (CA) is an ubiquitous metalloenzyme involved in many physiological processes such as pH regulation, electrolyte secretion, and several metabolic pathways [1]. In the kidney, isoforms II and IV are crucial to NaHCO_3_ reabsorption and acid secretion [2]. CA inhibition results in the abolition of NaHCO_3_ reabsorption in renal proximal tubules, leading to a strong diuretic effect and an increase in HCO_3_^−^, K^+^, and Na^+^ urinary excretion [3]. CA inhibitors (CAIs), led by dorzolamide, brinzolamide, and acetazolamide, are used in the treatment of many pathologies such as glaucoma, intracranial hypertension, altitude sickness, and epilepsy [4,5,6,7,8] CAIs are also used in sports for doping purposes, as they dilute urine samples and modify drug metabolism, tampering with urinalyses [9]. The World Anti-Doping Agency (WADA) classified dorzolamide, brinzolamide, and acetazolamide as “diuretic and masking agents” (S5 class), which are prohibited substances in sport at all times (in- and out-of-competition) [10]. The identification of these substances or their biomarkers in an athlete’s sample by a WADA-accredited laboratory can trigger adverse analytical findings (AAFs), which can lead to sanctions. Dorzolamide was identified in 116 AAFs reported to the WADA anti-doping administration and management system from 2016 to 2020, with a steady increase from 0.2% to 1.4% in the total AAFs (from 1.5% to 9.9% in the AAFs involving S5 substances) from 2017 to 2020. Brinzolamide was identified in 43 AAFs (0.2% of the total AAFs and 1.8% of the S5 AAFs), and acetazolamide was identified in 47 AAFs (0.3% of the total AAFs and 1.8% of the S5 AAFs) over the same period [11,12,13,14,15]. Inadvertent doping through dietary supplement use was reported for acetazolamide [16].

**Figure 1:**
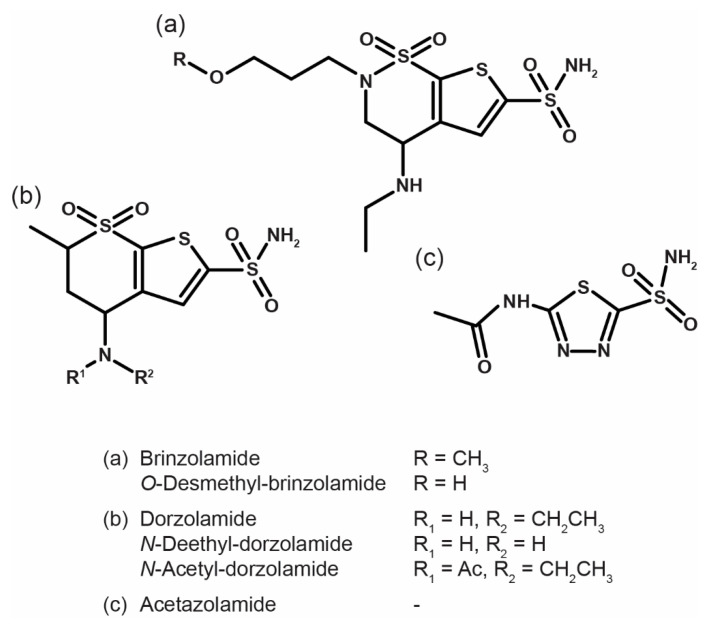
Skeletal formula of the carbonic anhydrase inhibitors and their metabolites included in the analytical method.

The identification of CAIs and/or their metabolites in urine and hair, for consumption history, is important to documenting exposure in analytical toxicology and doping [17]. In human beings, *N*-deethyl-dorzolamide is the predominant metabolite of dorzolamide in urine [18], while brinzolamide is mainly deactivated through oxidative *O*- and *N*-dealkylation [19]; although minor, acetazolamide *N*-acetyl, glucuronide, and cysteine conjugates are found in urine [20]. Dorzolamide, brinzolamide, and acetazolamide metabolites’ incorporation into hair is yet to be studied. We recently proposed an original method to simultaneously detect dorzolamide, brinzolamide, acetazolamide, and their major metabolites (Figure 1) in human urine and hair by ultra-high-performance liquid chromatography-tandem mass spectrometry (UHPLC-MS/MS) [21]. In the present study, we report the application of the method to estimate the differences in CAIs and/or their metabolites concentrations in urine and hair of patients undergoing dorzolamide, brinzolamide, or acetazolamide treatment and their relatives. Relatives were tested to evaluate the analytes’ potential for accidental exposure.

## 2. Materials and Methods

### 2.1. Chemicals and Reagents

Brinzolamide, dorzolamide (HCl salt), acetazolamide, *N*-acetyl-dorzolamide, *N*-deethyl-dorzolamide, and *O*-desmethyl-brinzolamide reference standards were obtained from LGC Standards (Teddington, Middlesex, UK). Deuterated internal standards (ISs) acetazolamide-d3 and brinzolamide-d5 were purchased from Cayman Chemical (Ann Arbor, MI, USA). All standards were stored at −20 °C until analysis.

LC-grade dichloromethane and LC-MS grade water, methanol, and formic acid were purchased from Sigma-Aldrich^®^ (Milano, Italy). A 5 mM ammonium acetate buffer was prepared with ≥99% purity ammonium acetate from Sigma–Aldrich^®^ in water and 0.1% formic acid. Acidic buffer M3^®^ (proprietary composition) was acquired from Comedical^®^ s.r.l. (Trento, Italy).

### 2.2. Calibrators and Quality Control Solutions

Brinzolamide, dorzolamide (base), acetazolamide, *N*-acetyl-dorzolamide, *N*-deethyl-dorzolamide, and *O*-desmethyl-brinzolamide stock solutions were prepared at 10, 1, and 0.1 μg/mL in methanol. A stock solution of deuterated standard was prepared at 1 μg/mL in methanol; the final IS concentration in drug-free spiked urine and hair was 5 ng/mL and 1 ng/mg, respectively. Based on an initial semi-quantitative analysis of the urine and hair samples, the calibration curves ranged from limit of quantification (LOQ) to 1000 ng/mL in the urine and from LOQ to 10 ng/mg in the hair for each analyte. High (HQCs), medium (MQCs), and low (LQCs) quality controls were prepared at 350, 87, and 31.5 ng/mL, respectively, in drug-free urine. HQCs, MQCs, and LQCs were prepared at 7.5, 3.5, and 0.5 ng/mg, respectively, in drug-free hair.

### 2.3. Sample Treatment

Samples were extracted as previously described [21].

Briefly, 100 μL urine was spiked with a 5 μL ISs working solution and vortexed. After adding 5 mL of 0.1% formic acid in 5 mM ammonium acetate buffer:0.01% formic acid in methanol (95:5, *v*/*v*), samples were vortexed and centrifuged. Supernatants (100 μL) were transferred into autosampler glass vials, prior to injection onto the chromatographic system. Injection volume was 3 μL.

Hair samples were washed twice with dichloromethane and dried under nitrogen at 45 °C. A 20 mg aliquot was finely cut (<5 mm) and spiked with 50 μL ISs working solution. After adding 500 μL M3^®^ reagent, tubes were vortexed and incubated at 100 °C for 1 h for complete hair hydrolysis. After cooling at room temperature, 200 μL was transferred into autosampler glass vials without further sample treatment, prior to injection onto the chromatographic system. Injection volume was 1 μL.

### 2.4. Ultra-High-Performance Liquid Chromatography-Tandem Mass Spectrometry (UHPLC-MS/MS) Analysis

Samples were analyzed as previously described [21] with a Waters^®^ Xevo^®^ TQ-S micro mass spectrometer (triple quadrupole) interfaced with an ACQUITY UPLC^®^ I-Class (Waters^®^; Milano, Italy) equipped with an electrospray ionization source operating in positive-ion mode. Data were acquired with MassLynx^®^ software version 4.1 from Waters^®^.

Separation was performed through an ACQUITY UPLC^®^ BEH C18 column (length: 50 mm, internal diameter: 2.1 mm, particle size: 1.7 μm) from Waters^®^ with a gradient mobile phase composed of 0.1 formic acid in 5 mM ammonium acetate buffer (A) and 0.01% formic acid in methanol (B) at 50 °C. Initial gradient conditions were 5% B held for 0.25 min; B was increased to 20% within 2.75 min, then 95% within 2 min; 95% B was held for 0.5 min, before returning to initial conditions within 0.1 min; re-equilibration time was 2.6 min. Total run time was 8 min at a flow rate of 0.35 mL/min.

Multiple-reaction monitoring (MRM) acquisition was used with two transitions for each analyte and IS. MS transitions were monitored as follows (quantification transition first): *m*/*z* 325.1 > 135.1 and *m/z* 325.1 > 199.0 for dorzolamide, *m/z* 330.1 > 135.1 and *m/z* 330.1 > 199.0 for dorzolamide-d5, *m/z* 297.1 > 135.1 and *m/z* 297.1 > 199.0 for *N*-deethyl-dorzolamide, *m/z* 367.1 > 88.1 and *m/z* 367.1 > 135.1 for *N*-acetyl-dorzolamide, *m/z* 384.0 > 217.1 and *m/z* 384.0 > 281.0 for brinzolamide, *m/z* 370.0 > 136.9 and *m/z* 370.0 > 181.0 for *O*-desmethyl-brinzolamide, *m/z* 223.1 > 73.3 and *m/z* 226.1 > 163.0 for acetazolamide, and *m/z* 226.1 > 73.3 and *m/z* 226.1 > 165.0 for acetazolamide-d3.

### 2.5. Method Validation

The method was validated following the standard practices for the validation of analytical assays in toxicology [22].

In urine, the method was linear for all the analytes within their calibration range. Accuracies were within 14.6% of target, while intra- and inter-assay precision were within 7.7% of target. Analytical recovery ranged from 81.0 to 98.1%, while matrix effect ranged from −21.2 to −3.0%. Limits of detection (LODs) and quantification (LOQs) were 0.11 and 0.38 ng/mL for dorzolamide, 0.07 and 0.24 ng/mL for *N*-deethyl-dorzolamide, 0.17 and 0.55 ng/mL for *N*-acetyl-dorzolamide, 0.02 and 0.07 ng/mL for brinzolamide, 0.35 and 1.16 ng/mL for *O*-desmethyl-brinzolamide, and 0.13 and 0.43 ng/mL for acetazolamide [21].

In hair, the method was linear for all the analytes within their calibration range. Accuracies were within 14.6% of target, while intra- and inter-assay precision were within 14.6% of target. Analytical recovery ranged from 96.5 to 99.0%, while matrix effect ranged from −22.0 to −3.4%. Limits of detection (LODs) and quantification (LOQs) were 0.01 and 0.02 ng/mg for dorzolamide, 0.01 and 0.04 ng/mg for *N*-deethyl-dorzolamide, 0.01 and 0.02 ng/mg for *N*-acetyl-dorzolamide, 0.02 and 0.06 ng/mg for brinzolamide, 0.05 and 0.15 ng/mg for *O*-desmethyl-brinzolamide, and 0.01 and 0.03 ng/mg for acetazolamide [21].

### 2.6. Samples from Patients and Relatives

A total of 88 patients undergoing treatment for glaucoma and high intraocular pressure, i.e., oral dorzolamide, brinzolamide, and/or acetazolamide, were recruited from the Eye Clinic (Polytechnic University of Marche, Ancona, Italy) during a routine medical checkup. A relative sharing the patient’s household was recruited for each patient (spouse or offspring). All the patients and their relatives were over the age of legal majority and gave informed consent prior to the study. The experiments were conducted in accordance with the Helsinki Declaration.

Two hair aliquots were collected with clean scissors at the occipital region of the nape of the neck, cutting close to the scalp. One aliquot was analyzed while the other was conserved for further investigations. Hair samples were stored at room temperature until analysis. Urine samples were collected separately in two sterile 10 mL plastic containers and stored at −20 °C until use.

### 2.7. Statistical Analysis

Descriptive analysis was conducted to describe the study cohort: mean and standard deviation (sd) or median and interquartile range (IQR) were used for quantitative variables according to their distribution. Absolute and percentage frequencies were used to summarize qualitative variables.

In order to evaluate statistical differences between the CAI concentrations detected in patients and the CAI concentrations detected in their relatives, the Student’s *t*-test or the Wilcoxon sum-rank test were used according to the variable distribution. Chi-square test was used to compare qualitative variables. The paired samples Wilcoxon sum-rank test was used to compare the concentration of dorzolamide and the concentrations of its related metabolites. Benjamini–Hochberg *p*-value adjustment method was applied. The significance level for all the analyses was set at *p* < 0.05. Statistical analyses were performed using open-source freeware R, version 4.1.0.

## 3. Results

A total of 88 patients undergoing CAI treatment for glaucoma and high intraocular pressure were recruited: 49 patients with dorzolamide, 29 with brinzolamide, 9 with acetazolamide, and 1 with both dorzolamide and acetazolamide. The distributions of each variable were asymmetric; hence, a non-parametric statistical approach was chosen. Patients were mainly males (72.73%) with a median age of 70 years (IQR: 62–79 years), while 61.36% relatives were female with a median age of 64 years (IQR: 52–76 years) (Table 1). Gender-related discrepancies in dorzolamide, brinzolamide, and acetazolamide pharmacokinetic profiles are unknown.

The concentrations of CAIs and metabolites in the urine and hair samples of patients undergoing treatment and their relatives are reported in the Supplementary Materials (Tables S1–S3).

Dorzolamide was detected in all urine samples and in 49 (98%) hair samples (9, 18.4% with concentration lower than LOQ), *N*-deethyl-dorzolamide in all urine and in 48 (96%) hair samples, in which 23 (47.9%) had a concentration lower than LOQ, while *N*-acetyl-dorzolamide was detected in 43 (86%) urine samples, in which 21 (48.8%) reported a concentration lower than LOQ; no *N*-acetyl-dorzolamide was detected in patients’ hair. The concentration of dorzolamide was significantly higher (*p* < 0.001) than the concentration of *N*-deethyl-dorzolamide both in patients’ urine and hair samples, and significantly higher (*p* < 0.001) than the *N*-acetyl-dorzolamide concentration in patients’ urine (Table 2). Dorzolamide was detected only in three (6%) relatives’ urine samples, in which one with concentration lower than LOQ, while *N*-deethyl-dorzolamide and *N*-acetyl-dorzolamide were not detected (Table 2 and Table S1).

In 29 patients under brinzolamide treatment, brinzolamide was detected in all urine samples, and in 28 (96.6%) hair samples, in which 14 (50%) reported a concentration lower than LOQ; *O*-desmethyl-brinzolamide concentrations were lower than LOQ in almost all urine samples (*n* = 28, 96.6%), while no trace of this metabolite was detected in hair; brinzolamide was detected in only one (3.4%) patients’ relative with a urinary concentration lower than LOQ.

In 10 patients under acetazolamide treatment, acetazolamide was detected in all urine samples and in only one hair sample. *N*-deethyl-dorzolamide, *N*-acetyl-dorzolamide, *O*-desmethyl-brinzolamide, and acetazolamide were not detected in relatives.

## 4. Discussion

We quantified CAIs and their metabolites in the urine and hair of patients undergoing treatment and their relatives. Acetazolamide human metabolism was recently assessed using in vitro hepatocyte incubations and in vivo urine and plasma samples from patients [20]. However, reference standards of acetazolamide major metabolites, i.e., *N*-acetyl-acetazolamide and acetazolamide-cysteine, are not yet available and, therefore, they could not be included in the present method.

In patients urine, dorzolamide was approximately 2 to 70 times more concentrated than the main metabolite, *N*-deethyl-dorzolamide, in all samples except for one (#19), consistent with previously published data [18]. Although *N*-acetyl-dorzolamide was detected in all the urine samples of patients under dorzolamide treatment, the concentration was above the LOQ only in less than half of the cases, making it an inadequate biomarker of consumption. *O*-Desmethyl-brinzolamide urinary concentration also was low compared to that of brinzolamide, and was above the LOQ in only one case (#5), as previously observed [19]. These results are not surprising considering CAI polarity and tropism for kidneys. However, urinary metabolite detection may be critical to document CAI use in doping, as the presence of the metabolites rules out sample tampering. In hair, parent drug concentrations were much higher than those of their metabolites, similar to blood concentrations, as xenobiotics are directly incorporated from the bloodstream into hair through their roots [18,19]. To the best of our knowledge, this is the first time that brinzolamide concentrations are reported in hair; *O*-brinzolamide was not detected. The detection of the drugs in hair helps document consumption history.

In 4 of 79 cases, brinzolamide or dorzolamide was detected in the urine of the relatives, although the concentrations were low compared to what was detected in actual patients. To rule out potential cross-contamination during the analysis, a different aliquot of the relatives’ positive samples were reanalyzed. To avoid false positive cases, WADA recommended the detection of at least 20 ng/mL acetazolamide or metabolites in urine to trigger an AAF in sport, subsequent to the detection of the substance as a contaminant in an athlete’s urine after consuming dietary supplements [16,23]. However, the detection of dorzolamide or brinzolamide in urine may be sufficient to trigger an AAF, potentially resulting in sanctions. In the present study, the patients’ relatives may have been accidentally exposed, although the manner of exposure cannot be elucidated. Setting cutoff concentrations of urinary dorzolamide and brinzolamide is therefore crucial to address doping cases. The analytes were not detected in the hair samples of patients’ relatives.

## 5. Conclusions

Dorzolamide, brinzolamide, and acetazolamide are regularly involved in doping, and the detection of these substances and their metabolite biomarkers in biological matrices is necessary to document consumption. Furthermore, it is necessary to distinguish therapeutic use from doping use. We reported the concentrations of dorzolamide, brinzolamide, acetazolamide, and their metabolites in the urine and hair of 88 patients undergoing long-term treatment for ocular hypertension or glaucoma. For the first time, we report brinzolamide concentrations in hair, which can help assess an individual’s exposure history. Additionally, we detected dorzolamide and brinzolamide in the urine of three patients and one relatives of a patient, respectively, indicating that contamination is possible, although with a low probability. Cutoff concentrations of urinary dorzolamide and brinzolamide are necessary to rule out false positives due to contamination or passive exposure.

## Figures and Tables

**Table 1:** Patient and relative characteristics according to patients’ treatment.

Treatment		*n* (%)	Age [Year, Median (IQR)]	Sex [Male, *n* (%)]
**Dorzolamide**	Patients	50 (56.8)	70 (63; 80)	39 (78)
	Relatives	50 (56.8)	64 (46; 77)	19 (38)
	*p*		0.05 ^1^	<0.01 ^2^
**Brinzolamide**	Patients	29 (33)	70 (61; 79)	21 (72.4)
	Relatives	29 (33)	61 (55; 74)	10 (34.5)
	*p*		0.06 ^1^	0.01 ^2^
Acetazolamide *	Patients	9 (10.2)	67 (59; 75)	4 (44.4)
	Relatives	9 (10.2)	67 (55; 75)	5 (55.6)
	*p*		0.97 ^1^	0.99 ^2^

*p* refers to the *p*-value of ^1^ Wilcoxon sum-rank test and ^2^ Chi-square test; IQR, Interquartile range. * The patient with both dorzolamide and acetazolamide treatment was included in dorzolamide group.

**Table 2:** Comparisons of substances and metabolites concentrations between patients and their relatives.

	*n* *	*n*°	Patients	Relatives	*p*
**Urine**					
Dorzolamide [ng/mL, median (IQR)]	50	0	245 (116; 501) #	0 (0; 0)	<0.001
*N*-Deethyl-dorzolamide [ng/mL, median (IQR)]	50	0	81.1 (35.9; 125.3) #	0 (0; 0)	<0.001
*N*-Acetyl-dorzolamide [ng/mL, median (IQR)]	22	21	0.77 (0.64; 0.84) #	0 (0; 0)	<0.001
Brinzolamide [ng/mL, median (IQR)]	29	0	38.9 (20.4; 79.2)	0 (0; 0)	<0.001
*O*-Desmethyl-brinzolamide [ng/mg, median (IQR)]	1	28	-	0 (0; 0)	
Acetazolamide [ng/mL, median (IQR)]	10	0	72.8 (20.7; 437.3)	0 (0; 0)	
**Hair**					
Dorzolamide [ng/mg, median (IQR)]	40	9	0.48 (0.1; 0.98) #	0 (0; 0)	<0.001
*N*-Deethyl-dorzolamide [ng/mg, median (IQR)]	26	23	0.07 (0.06; 0.08) #	0 (0; 0)	<0.001
*N*-Acetyl-dorzolamide [ng/mg, median (IQR)]	0	0	-	-	
Brinzolamide [ng/mg, median (IQR)]	14	14	0.4 (0.13; 1.95)	0 (0; 0)	<0.001
*O*-Desmethyl-brinzolamide [ng/mg, median (IQR)]	0	0	0 (0; 0)	0 (0; 0)	
Acetazolamide [ng/mg, median (IQR)]	1	9	-	0 (0; 0)	0.05

*n* *, number of patients in which the concentration was > LOQ; *n*°, number of patients in which the concentration was >0 and <LOQ; *p* refers to the Wilcoxon sum-rank test; IQR, Interquartile range; LOQ, limit of quantification. # comparison of concentrations of dorzolamide and its related metabolites: paired samples Wilcoxon sum-rank test *p* < 0.001.

## Data Availability

Data are contained within the article.

## Supplementary Materials

The following supporting information can be downloaded at: https://www.mdpi.com/article/10.3390/biology11101379/s1, Table S1: Concentrations of dorzolamide and metabolites in urine and hair samples of patients undergoing dorzolamide treatment, and their relatives; Table S2: Concentrations of brinzolamide and its metabolite in urine and hair samples of patients undergoing brinzolamide treatment, and their relatives; Table S3: Concentrations of acetazolamide in urine and hair samples of patients undergoing acetazolamide treatment, and their relatives.
